# Simultaneous ASFV and Haptoglobin Detection by Duplex qPCR Enables Pre-Viremia Diagnosis of African Swine Fever

**DOI:** 10.3390/v17111444

**Published:** 2025-10-30

**Authors:** Yun Bao, Shimin Gao, Shuang Li, Yijie Liu, Fei Gao, Liwei Li, Wu Tong, Changlong Liu, Yanjun Zhou, Yifeng Jiang

**Affiliations:** 1 College of Veterinary Medicine, Shanxi Agricultural University, Jinzhong 030801, China; 2 Shanghai Veterinary Research Institute, Chinese Academy of Agricultural Sciences, Shanghai 200241, China; 3 Jiangsu Co-Innovation Center for the Prevention and Control of Important Animal Infectious Diseases and Zoonoses, Yangzhou University, Yangzhou 225009, China

**Keywords:** HP, ASFV, early detection, Duplex qPCR

## Abstract

African swine fever (ASF), caused by African swine fever virus (ASFV), has inflicted severe economic losses on China’s pig industry. Existing ASFV nucleic acid detection methods struggle to identify infected pigs in the pre-viremic stage, especially for recently emerged recombinant ASFV strains that exhibit delayed clinical symptoms and prolonged virus shedding, posing great challenges to ASF prevention and control. To fit the problem, this study established a TaqMan duplex quantitative polymerase chain reaction (qPCR) assay targeting the ASFV p72 gene and porcine Hp gene for early diagnosis of ASFV infection. The qPCR reaction system (20 μL) and conditions were optimized and showed high sensitivity, with detection limits of 1.42 × 10^1^ copies/μL for Hp and 2.23 × 10^1^ copies/μL for ASFV, as well as excellent specificity and reproducibility. Serum cDNA samples from pigs infected with virulent or recombinant ASFV strains were tested, and the result showed that Hp was detectable as early as 1 day post-infection (DPI), however ASFV remained undetectable until 3DPI. Then cDNA samples from cohabitation infection were tested and 80% samples were Hp-positive, although ASFV test was negative.In conclusion, this duplex qPCR assay for simultaneous detection of Hp and ASFV enables pre-viremia diagnosis of ASF, providing a valuable tool for early screening of ASFV-infected pigs.

## 1. Introduction

African swine fever (ASF) has caused devastating economic losses to China’s pig industry, since it emerged in 2018 [1,2,3]. ASFV infections cause nearly 100% mortality among pigs, and there is no vaccine or drug that can effectively prevent and control ASF [4]. Currently, pig farms can rely on biosecurity measures to prevent and control ASF, especially through the early diagnosis of infected pigs to avoid large-scale infections. Pigs infected with the Georgia strain develop clinical symptoms, such as high fever, quickly, and pig farms can eliminate the infected pig through the “test-and-cull” method [4]. However, the recombinant strains which emerged recently cause delayed clinical symptoms, leading to a long period of virus shedding in infected pigs, before they were detected [5,6,7]. Most of the ASFV nucleic acid detection methods are difficult to identify in pigs in the early stage of infection that have not yet developed viremia. As a result, once the recombinant strain infection occurs, the pig farms need to conduct “test-and-cull” operations continuously, which not only increase costs but also make it difficult to fully control ASFV infection [8,9]. Therefore, a method that can detect infected pigs in the earlier stages of ASFV infection is urgently needed [10].

Haptoglobin (Hp) is an acidic glycoprotein found in the α^2^-globulin fraction of serum [11]. It is primarily synthesized and metabolized in the liver, and is widely distributed in the serum and other body fluids of mammals [12]. Furthermore, as an important acute-phase protein in pigs, Hp functions as a marker for inflammatory diseases and is involved in processes such as antibacterial activity and immune regulation [13]. As a nonspecific inflammatory biomarker with significant physiological activity, Hp exhibits a rapid concentration increase following pathological damage in animals within 24 to 48 h, and returns to normal quickly once tissue recovery is achieved [14]. Its serum concentration correlates with disease severity and prognosis, as evidenced by a 23.9-fold increases in porcine circovirus type 2 infections [15].

## 2. Materials and Methods

This study established a TaqMan dual-fluorescence quantitative PCR (qPCR) method targeting ASFV p72 gene and Hp gene for the rapid diagnosis of pigs infected by ASFV in the early stages. The standard plasmids for Hp (NM_214000.2) and ASFV p72 gene (OM966719.1) used in this study were synthesized by Sangon Biotech (Shanghai, China) Co., Ltd. [16]. After measuring the concentration of the standard plasmids, the initial copy numbers were calculated as 1.42 × 10^10^ copies/μL for Hp and 2.23 × 10^10^ copies/μL for ASFV. Subsequently, the standard plasmids were diluted in a 10-fold to gradient eight dilution from 1.42 × 10^1^ to 1.42 × 10^8^ for Hp and 2.23 × 10^1^ to 2.23 × 10^8^ for ASFV, respectively, and reactions were performed using a LightCycler^®^96 Real-Time PCR System, manufactured by Roche (Basel, Switzerland). Primers and probes were designed based on the conserved sequences of Hp and ASFV p72 gene. For Hp detection, the primers and probe were designed as follows: Forward primer, 5′-GAAGTATGTCATGCTGCCGGTG-3′; Reverse primer, 5′-GAAGGTGTGCTCGTTCAGGATG-3′; Probe, 5′6-FAM-CAGTACTACGAAGGCAGCACCGTG-3′BHQ1; and the amplified fragment size was 122 bp. For ASFV detection, the primers and probe were designed as follows: Forward primer, 5′-GCTATTCCCTCAGTATCCATTCC-3′; Reverse primer, 5′-AAACGTGACTGGCGTACAA-3′; Probe, 5′-HEX-TCGGCGAGCGCTTTATCACCATAA-3′BHQ1; and the amplified fragment size was 113 bp. Subsequently, we optimized the qPCR reaction system: the concentrations of both primers and probes were diluted to 10 μmol/L. We selected 0.4, 0.5, 0.6, and 0.7 μL of primers, along with 0.4, 0.6, and 0.8 μL of probes, and used three temperatures (56 °C, 58 °C, and 60 °C) as annealing temperatures for combinatorial testing. 

## 3. Results

**Figure 1:**
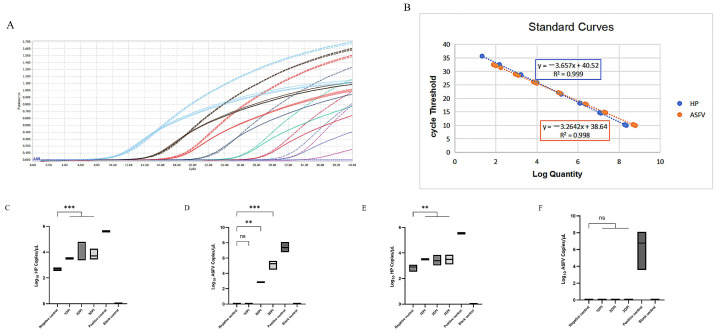
Establishment and evaluation of a dual TaqMan qPCR assay for African Swine Fever Virus and Haptoglobin. (**A**): Duplex fluorescent quantitative PCR amplification curve. Dashed lines represent real-time fluorescence amplification curves of ASFV standard samples from 2.23 × 10^1^–2.23 × 10^8^ copies; solid lines represent real-time fluorescence amplification curves of Hp standard samples from 1.42 × 10^1^–1.42 × 10^8^ copies; (**B**): Standard curve of the duplex fluorescent quantitative PCR method. The dual qPCR assay was conducted using the established method to test samples collected at 1–3 DPI from pigs infected with either the virulent or recombinant ASFV strain; (**C**): Hp detection results in virulent-strain-infected group (days 1–3); (**D**): ASFV detection results in virulent-strain-infected group (days 1–3); (**E**): Hp detection results in recombinant-strain-infected group (days 1–3); (**F**): ASFV detection results in recombinant-strain-infected group (days 1–3). Negative controls consisted of the serum cDNA from healthy pigs, positive controls were the cDNA of wild-type ASFV and recombinant strain, and blank controls were dd H_2_O. Time point with single “ns” above the black line means there was no significant difference among the groups. ** means *p* < 0.01, *** means *p* < 0.001.

Based on factors such as cycle threshold (Ct value), fluorescence intensity, and reaction cost, the optimal reaction system and conditions were determined as follows: The 20-μL duplex qPCR system consisted of 10 μL QPS-101 THUNDERBIRD Probe qPCR Mix, 0.5 μL of each Hp primer, 0.6 μL of the Hp probe, 0.5 μL of each ASFV primer, 0.7 μL of the ASFV probe, 2 μL of test template, and nuclease-free water to make up a total volume of 20 μL. The fluorescent quantitative PCR conditions were set as follows: an initial denaturation step at 95 °C for 30 s, followed by 40 cycles of denaturation at 95 °C for 5 s and annealing at 60 °C for 30 s. Standard curves were constructed by plotting the logarithm (base 10) of the initial copy number of the standard plasmids on the horizontal axis and the Ct value on the vertical axis. The detection limits for Hp and ASFV were 1.42 × 10^1^ copies/μL and 2.23 × 10^1^ copies/μL, respectively, with good linearity (Figure 1A,B). The optimized method was used to detect nucleic acids of common swine virus diseases, including Porcine Reproductive and Respiratory Syndrome Virus (PRRSV), Porcine Circovirus Type 2 (PCV2), and Classical Swine Fever Virus (CSFV), as well as various acute-phase proteins such as C-reactive protein (CRP) and Transthyretin (TTR). The results demonstrated that this method exhibits high specificity and excellent reproducibility.

The cDNA samples (extracted from the serum of infected pigs) of the ASFV virulence (GZ2018, ON263123.1 and Pig/HLJ/18, MK333180.1) and recombinant strains (JS/LG/21, OQ504956) used for detection of Hp and ASFV were kindly provided by Professors Guihong Zhang and Dongming Zhao (Table 1). The results showed that for the virulence strain, Hp could be detected one day post-infection (DPI) (Figure 1C); however, ASFV could not be detected until 2DPI (Figure 1D), with only one sample from 2 DPI being positive (Table 1). For the recombinant strain, Hp was also detectable at 1 DPI (Figure 1E), whereas ASFV remained undetectable from 1 to 3 DPI (Figure 1F). Subsequently, this method was applied to detect cDNA samples collected from pigs either artificially or naturally infected with the recombinant ASFV strain, 6-9 days post-infection during the cohabitation challenge trial. The results showed that 80% of the samples in the cohabitation-infected group exhibited a significant increase in Hp, whereas none of the samples were detected as ASFV positive (Table 1). The test results are consistent with the background of the samples.

## 4. Discussion

African swine fever virus belongs to the genus Asfivirus within the family Asfarviridae. Clinically, it primarily causes symptoms in pigs such as high fever, loss of appetite, lethargy, weakness, hemorrhagic signs, and respiratory distress, with a mortality rate of up to 100% in susceptible pig populations [17,18]. Due to its severe impact, China has classified it as a Category I animal disease. In early 2021, a naturally recombinant strain of ASFV was isolated in China, and the emergence of recombinant strains has further complicated the prevention and control of ASF [19]. Pig farms use biosecurity measures to prevent and control African Swine Fever (ASF). They control the spread of the virus by detecting and culling pigs infected with the African Swine Fever Virus (ASFV). Most of the existing ASFV antigen detection methods are nucleic acid tests, which have high sensitivity. The sensitivity of the detection method we established is comparable to that of other methods. However, the difficulty in clinical prevention and control of ASF lies in how to identify infected pigs before viremia occurs, thereby controlling the spread of the virus through the “test-and-cull” method. In the early stages of disease infection, the concentration of Hp can increase dramatically even before viremia, this makes HP a characteristic marker of early infection [20]. Our research results showed that Hp can be detected in cDNA samples as early as 1DPI, 24 to 48 h earlier than the ASFV nucleic acid detection method, more over Hp method can detect more potentially infected pigs than the ASFV nucleic acid detection method, this indicates that Hp can serve as an indicator for ASFV infection.

Although Hp has the potential to indicate African swine fever virus (ASFV) infection, it should be noted that as an acute-phase protein, infections caused by other viruses (such as porcine circovirus type 2 and porcine reproductive and respiratory syndrome virus) or bacteria can also lead to an increase in Hp concentration [12,21,22,23]. Therefore, in clinical practice, the determination of ASFV infection using the Hp index must be combined with the actual situation and the results of ASFV nucleic acid detection. For example, when the health status of the pig herd is stable, if the surrounding ASF epidemic is severe, this method can be used as an early warning for ASFV infection; in emergency situations where ASFV infection occurs in the pig herd, this method can also be used to screen and cull potentially infected pigs, so as to reduce possible economic losses.

In summary, this study has established a dual-fluorescence quantitative PCR method for Hp and ASFV, which has good sensitivity and specificity. The method could be used for early screening of ASFV-infected pigs under specific conditions.

## Figures and Tables

**Table 1:** Results of double fluorescence quantitative PCR detection.

	Virulence Strain			Recombinant Strain			Artificial Infection	Cohabitation Natural Infection
	1DPI	2DPI	3DPI	1DPI	2DPI	3DPI		
Hp	6/6	14/14	6/6	6/6	6/6	6/6	4/4	16/20
ASFV	0/6	1/14	3/6	0/6	0/6	0/6	4/4	0/20

## Data Availability

The original contributions presented in this study are included in the article. Further inquiries can be directed to the corresponding author.
